# Immortalized Human hTert/KER-CT Keratinocytes a Model System for Research on Desmosomal Adhesion and Pathogenesis of Pemphigus Vulgaris

**DOI:** 10.3390/ijms20133113

**Published:** 2019-06-26

**Authors:** Benedikt Beckert, Francesca Panico, Robert Pollmann, Rüdiger Eming, Antje Banning, Ritva Tikkanen

**Affiliations:** 1 Institute of Biochemistry, Medical Faculty, University of Giessen, Friedrichstrasse 24, 35392 Giessen, Germany; 2 Department of Dermatology and Allergology, Philipps-Universität Marburg, Baldingerstraße, 35043 Marburg, Germany

**Keywords:** desmosome, desmoglein, flotillin, cell-cell adhesion, Pemphigus vulgaris, blistering disease, dermatology, epidermis

## Abstract

Pemphigus Vulgaris is an autoimmune disease that results in blister formation in the epidermis and in mucosal tissues due to antibodies recognizing desmosomal cadherins, mainly desmoglein-3 and -1. Studies on the molecular mechanisms of Pemphigus have mainly been carried out using the spontaneously immortalized human keratinocyte cell line HaCaT or in primary keratinocytes. However, both cell systems have suboptimal features, with HaCaT cells exhibiting a large number of chromosomal aberrations and mutated p53 tumor suppressor, whereas primary keratinocytes are short-lived, heterogeneous and not susceptible to genetic modifications due to their restricted life-span. We have here tested the suitability of the commercially available human keratinocyte cell line hTert/KER-CT as a model system for research on epidermal cell adhesion and Pemphigus pathomechanisms. We here show that hTert cells exhibit a calcium dependent expression of desmosomal cadherins and are well suitable for typical assays used for studies on Pemphigus, such as sequential detergent extraction and Dispase-based dissociation assay. Treatment with Pemphigus auto-antibodies results in loss of monolayer integrity and altered localization of desmoglein-3, as well as loss of colocalization with flotillin-2. Our findings demonstrate that hTert cells are well suitable for studies on epidermal cell adhesion and Pemphigus pathomechanisms.

## 1. Introduction

Pemphigus vulgaris (PV) and Pemphigus foliaceus (PF) are blistering autoimmune diseases of the skin and/or oral mucosa that result from loss of cell-cell adhesion of the epidermal/mucosal cells. In Pemphigus, autoantibodies against desmosomal components, mainly desmoglein-3 (Dsg3) and -1 (Dsg1) cause disruption of desmosomal cell adhesion, resulting in blistering of the epidermis and/or mucosa (for a review, please see [1]). The molecular events in keratinocytes that result in the loss of desmosomal cell adhesion have been actively studied for a number years. Two major mechanisms have been suggested, one postulating that steric hindrance due to the antibody binding to the extracellular domains of desmogleins breaks the desmosomes apart and causes acantholysis. On the other hand, it has been shown that pathogenic antibodies against desmogleins also induce active intracellular signaling events, which may indirectly result in loss of desmosomal cell adhesion. The molecular mechanisms of desmosomal adhesion and signaling in Pemphigus have recently been reviewed by Spindler and Waschke et al., and we would like to refer the readers to these reviews for a more detailed description [2,3]. 

Research aiming at understanding the molecular mechanisms in Pemphigus have mainly been carried out using either the spontaneously immortalized human keratinocytes, HaCaT cells (Human adult high Calcium low Temperature, [4]) or primary human or mouse keratinocytes. Most studies have used mainly monolayer cultures of these cells, but both HaCaT cells and primary keratinocytes can also be grown in 3-dimensional (3D) culture systems with an air-liquid interface, resulting in formation and differentiation of a multilayer tissue that is highly equivalent to epidermis. However, both cell systems have their drawbacks for the research, as summarized below and in Table 1. 

HaCaT cells are spontaneously immortalized human epidermal keratinocytes that were isolated from a skin region surrounding a cancerous lesion [4]. This cell line has been well studied and is favored by many researchers as it is easy and cheap to cultivate, virtually immortal, and can be differentiated in 3D cultures to form an epidermal tissue layer. However, HaCaT cells are pre-lesional and exhibit a mutated, inactive p53 protein and numerous passage dependent aberrations in chromosome numbers [4,5]. 

In addition to HaCaT cells, primary human keratinocytes are frequently used to study epidermal cell-cell adhesion and pemphigus pathogenesis. Although primary keratinocyte cultures well recapitulate their epidermal origin and are thus more “natural” keratinocytes than immortal cell lines, they also have several disadvantages. Primary keratinocytes can only be cultured for a very limited number of passages. Thus, they have to be re-purchased and re-expanded quite frequently to retain viable cultures, making their culture very expensive, as they also require special culture media. The limited supply also makes it difficult to perform certain experiments that require large amounts of material, such as isolation of membrane raft domains that contain desmosomal proteins. Furthermore, the source of the cells and the underlying genotype varies over time, which may result in reproducibility issues in some experiments. An additional disadvantage of primary cells is also that they are unsuitable for certain genetic modifications such as CRISPR/Cas-mediated knockout of genes, due to their limited lifetime. Therefore, a cell system that can more easily be manipulated and exhibits features of normal keratinocytes, but with an immortal growth, is highly desirable. 

hTert cells were originally derived from human foreskin keratinocytes and immortalized by expression of human telomerase and mouse Cdk4 [6,7]. They show typical features of basal epidermal keratinocyte stem cells, including expression of keratin 5 and p63. When grown in 3D organotypic cultures, hTert cells form an epidermis that well resembles that formed by primary keratinocytes under similar conditions [6]. Proper differentiation of hTert-immortalized keratinocytes in such cultures has been demonstrated by expression of e.g., involucrin and loricrin [6,8]. 

Although hTert cells also require a special keratinocyte culture medium for growth, they are not dependent on special coating of the culture surface, but grow on normal plastic surface. These cells exhibit a trisomy of chromosome 5 and 3–4 copies of chromosome 20, but these changes represent stable alterations of the karyotype that do not vary over time. Despite these chromosomal aberrations, hTert cells show a calcium dependent differentiation and expression of differentiation markers such as involucrin [6]. Thus, the chromosomal changes observed in this cell line do not appear to impair their differentiation, although we cannot completely exclude that minor differences to primary keratinocytes might be present. Therefore, hTert cells provide a model system that is genetically stable and identical over time, avoiding problems that may be caused by varying genotype in the primary keratinocytes or chromosomal aberrations and mutations in HaCaT cells. Since the hTert cell line is immortal, also experiments that are based on genome editing, such as CRISPR mediated knockouts, or stable protein expression are feasible. In addition, the supply is virtually unlimited, and experiments requiring high amounts of material are also possible. 

We have here studied the suitability of the immortalized human keratinocyte cell line hTert/KER-CT as a model system for studies on desmosomal adhesion and Pemphigus pathogenesis. We show that these cells express desmogleins in a calcium dependent manner, whereas the expression of flotillins, which we have shown to interact with Dsg3 [9], is calcium independent. However, flotillin-2 (Flot2) is only detected at the plasma membrane and colocalizes with Dsg3 under high calcium. Treatment of hTert cells grown under high calcium with IgG purified from PV patients (PV IgG) or with an anti-Dsg3 antibody (AK23, [10]) results in an aberrant Dsg3 staining and loss of colocalization with Flot2. In addition, desmosomal adhesion is weakened by PV IgG and AK23 in the dissociation assay. In summary, our data show that hTert cells provide a versatile and immortal cell system that is suitable for studies of desmosomal adhesion. 

## 2. Results

We here aimed at testing the suitability of the recently generated, commercially available human keratinocyte cell line hTert (KER-CT, ATCC CRL-4048) as a model system to study the mechanisms of loss of desmosomal cell adhesion in Pemphigus. These cells represent human foreskin keratinocytes that have been immortalized by stable expression of the human telomerase and the mouse Cdk4 kinase. These cells have been shown to express basal keratinocyte markers and to form a characteristic epidermis in 3D cultures [8]. 

**Figure 1:**
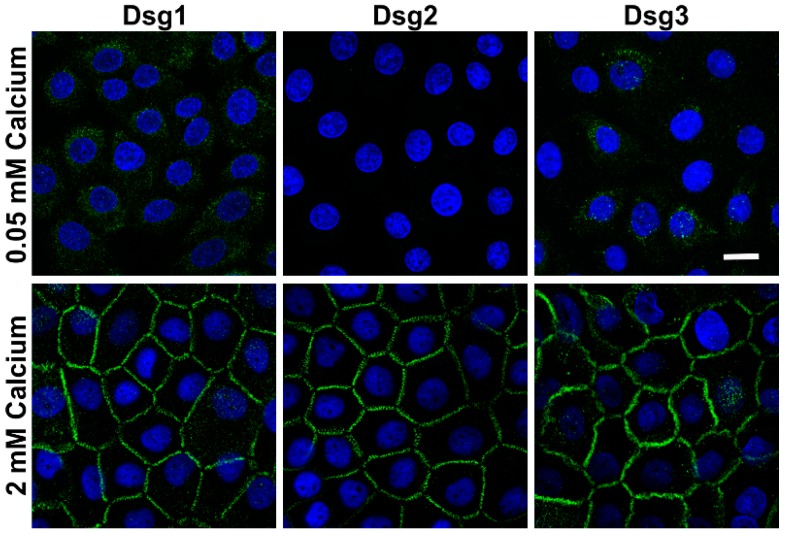
Effect of calcium concentration on the localization of desmogleins in human telomerase reverse transcriptase (hTert) cells. The cells were grown on coverslips in Keratinocyte growth medium (KGM) with 0.05 mM calcium for at least four days, and then shifted to 2 mM calcium for 24 h. After methanol (MeOH) fixation, the cells were stained with anti-desmoglein (Dsg) antibodies and fluorochrome coupled secondary antibodies (anti-mouse Alexa488, green). The coverslips were mounted in a mounting medium with 4′,6-diamidino-2-phenylindole (DAPI, blue) to visualize nuclei. *n* = 4 independent experiments, scale bar 20 µm.

Calcium is essential for the maturation of desmosomes and establishment of firm desmosomal adhesion [11,12,13]. We first characterized the expression and localization of Dsg1, 2 and 3 in hTert cells by immunofluorescence staining after culturing the cells on coverslips for several days in a growth medium containing 0.05 mM calcium. The expression of the three desmogleins was found to be very low and mostly perinuclear (Figure 1, upper row). However, when the cells were switched to 2 mM calcium for 24 h, the expression of desmogleins was greatly increased, and they became localized at the cell-cell borders (Figure 1, lower row). 

**Figure 2:**
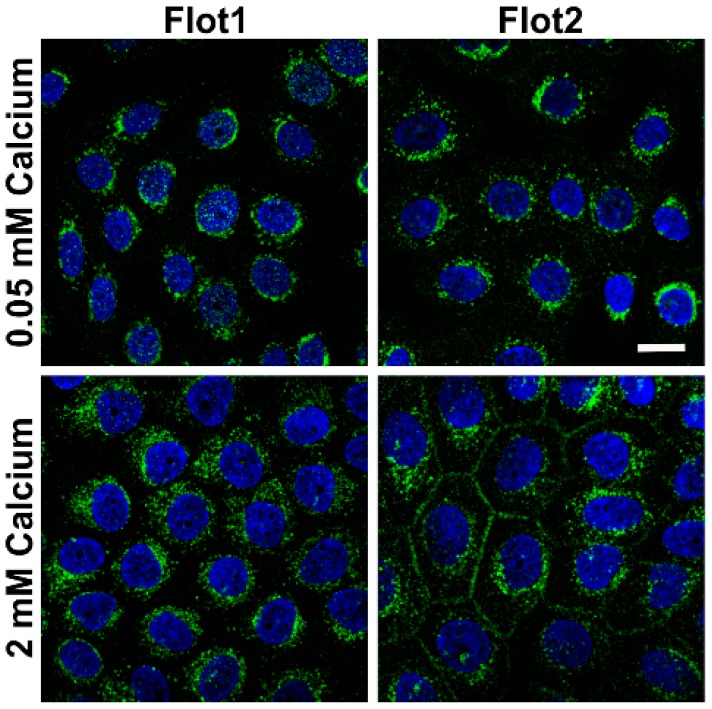
Effect of calcium concentration on the localization of flotillins in hTert cells. The cells were grown on coverslips in KGM with 0.05 mM calcium for at least four days, and then shifted to 2 mM calcium for 24 h. After MeOH fixation, the cells were stained with anti-flotillin antibodies and fluorochrome coupled secondary antibodies (anti-mouse Alexa488, green). The coverslips were mounted in a mounting medium with DAPI (blue) to visualize nuclei. *n* = 4 independent experiments, scale bar 20 µm.

**Figure 3:**
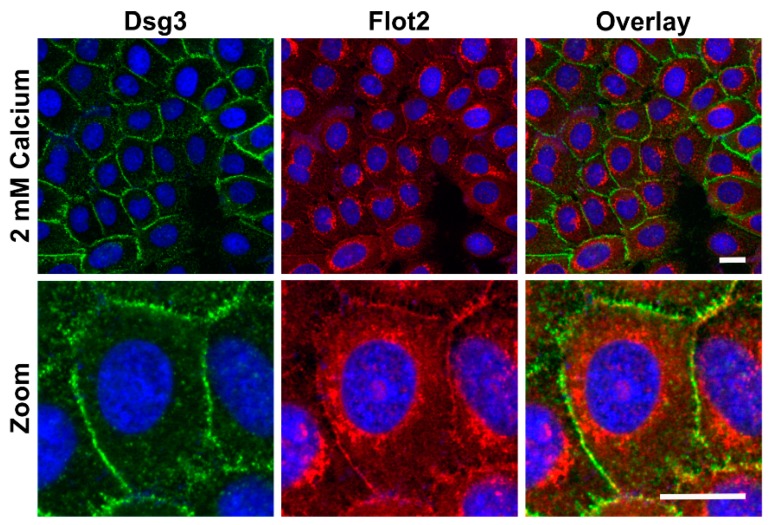
Colocalization of desmogleins and flotillin-2 upon 2 mM calcium in hTert cells. The cells were grown on coverslips in KGM with 0.05 mM calcium for at least four days, and then shifted to 2 mM calcium for 24 h. After MeOH fixation, the cells were stained with anti-Dsg3 (green) and anti-Flot2 (red) antibodies and fluorochrome coupled secondary antibodies (anti-mouse Alexa488 and anti-rabbit Alexa546). The coverslips were mounted in a mounting medium with DAPI. *n* = 3 independent experiments, scale bar 20 µm.

We also tested if the expression of flotillins, which we have shown to be associated with desmosomal proteins [9] would be altered in a similar way. In the low calcium medium, flotillin-1 (Flot1) and Flot2 were also found to be localized in the perinuclear region, whereas especially Flot2 partially localized at the cell-cell borders upon 2 mM calcium (Figure 2). Under 2 mM calcium, Flot2 was found to partially colocalize with Dsg3 at the cell-cell borders (Figure 3), similarly to what we have shown with HaCaT keratinocytes [9]. 

**Figure 4:**
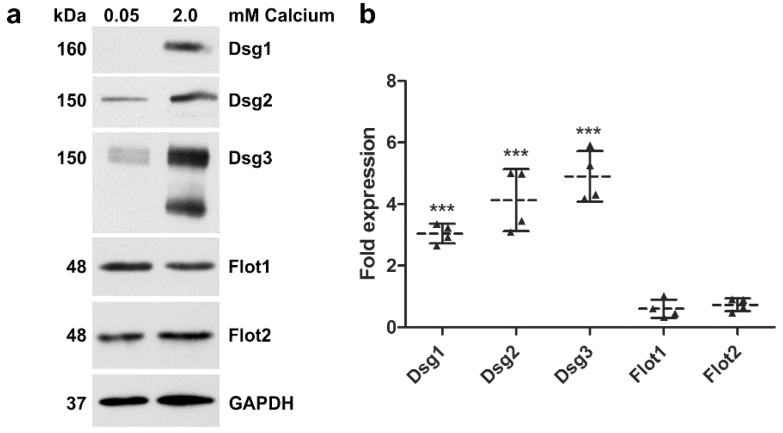
Effect of calcium on the expression of desmogleins and flotillins in hTert cells. The cells were grown in KGM with 0.05 mM calcium, and one plate was treated with 2 mM calcium for 24 h. (**a**) After cell lysis, equal protein amounts of the lysates were loaded onto gel and the expression of the indicated proteins was analyzed by Western blot. Glyceraldehyde 3-phosphate dehydrogenase (GAPDH) was used as a loading control; (**b**) quantification of the bands was performed with Quantity-One software. The expression signal in the 0.05 mM sample was set to one, and the relative fold expression levels in the 2 mM samples are shown as a scatter plot. Statistical analysis was done using one-way analysis of variance (ANOVA) with Bonferroni’s multiple comparison test. Statistically significant differences, as compared to the respective 0.05 mM sample, are indicated by *** *p* < 0.001. *n* = 4 independent experiments. Dotted line: Mean of samples, solid lines: SD.

**Figure 5:**
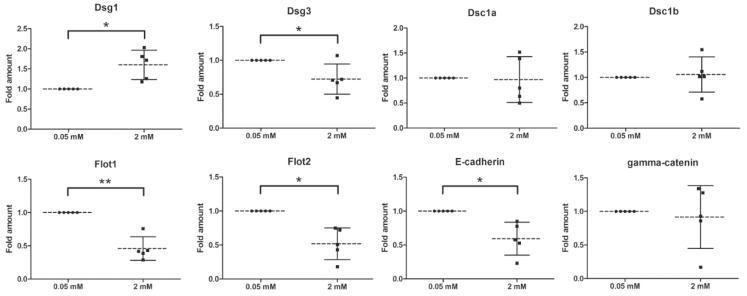
Effect of calcium concentration on the mRNA levels of adhesion proteins in hTert cells. The cells were grown in KGM with 0.05 mM calcium, then treated or not with 2 mM calcium for 24 h. RNA was isolated from the cells and quantitative real-time PCR was performed using the primers shown in Table 1. The ∆Ct-method was used to quantify the PCR products. The mean of the reference genes *RPL13a*, *GAPDH*, *Ywhaz* and *B2M* was used for normalization. The signal in the 0.05 mM calcium sample was set to one, and the relative fold amount in the 2 mM sample was calculated. For statistical analysis, the unpaired Student’s t-test was used. Statistically significant differences are indicated by * *p* < 0.05, and ** *p* < 0.01. *n* = 5 independent experiments. Dotted line: Mean of samples, solid lines: SD.

Western blot analysis (Figure 4a) and quantification (Figure 4b) of the expression of desmogleins in hTert cells showed that 2 mM calcium highly significantly induced the expression of Dsg1, 2 and 3, about 3- to 5-fold, whereas the expression of flotillins was either not significantly altered or was even slightly reduced as compared to 0.05 mM calcium (Figure 4, full Western blot slices are shown in Supplementary Materials). Quantitative real-time PCR analysis of the mRNA levels of several cell adhesion proteins demonstrated that the mRNAs of Dsg1, Desmocollin-1 isoforms a and b (Dsc1a and Dsc1b) and γ-catenin/plakoglobin were not significantly altered upon calcium switch, whereas the mRNAs of Dsg3, Flot1, Flot2 and E-cadherin were significantly lower in 2 mM calcium (Figure 5). These data demonstrate that the observed increase in the expression of desmogleins upon 2 mM calcium is not primarily due to transcriptional regulation.

**Figure 6:**
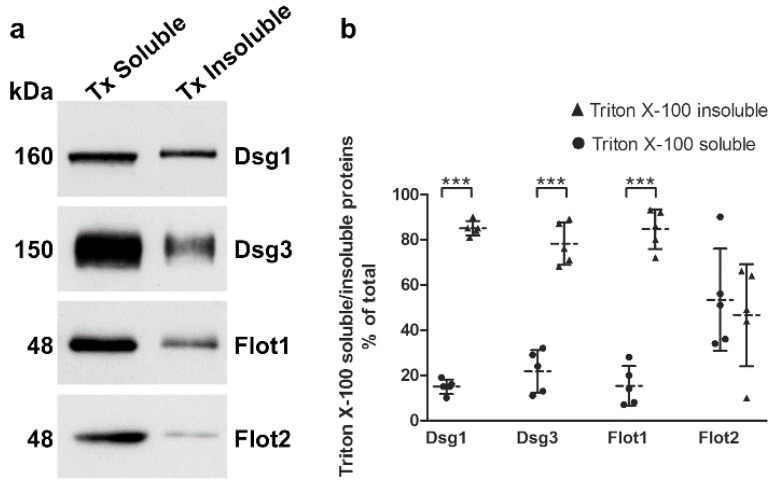
Detergent solubility of desmogleins and flotillins in hTert cells. The cells were grown in KGM with 2 mM calcium for 24 h and a sequential detergent extraction was performed, resulting in two pools with detergent soluble and insoluble proteins. (**a**) Western blot analysis of these fractions was used to detect Dsg1, Dsg3, Flot1 and Flot2; (**b**) quantification of the gel bands was performed with the Quantity-One software, and the percentage of the total cellular protein was calculated taking into account that triton X-100 (TX) soluble bands represent a higher relative percentage of the respective fraction than the TX insoluble bands. For statistical analysis, unpaired Student’s *t*-test was used. Statistically significant differences are indicated by *** *p* < 0.001. *n* = 5 independent experiments. Dotted line: Mean of samples, solid lines: SD.

Desmogleins that are present in mature desmosomes are insoluble in detergents like Triton X-100, and a large fraction of desmogleins is even present in membrane rafts [14,15]. The cellular desmoglein pool can be divided into desmosomal (detergent insoluble) and extradesmosomal (detergent soluble) pools by sequential detergent extraction (see e.g., [9,15]). We cultured hTert cells in 2 mM calcium for 24 h and performed extraction with Triton X-100, as described previously [9,15]. The detergent soluble proteins are present in the supernatant, whereas the detergent insoluble pellet fractions (desmosomal pool) were extracted and solubilized with SDS, and both fractions were analyzed by Western blot (Figure 6a). More than 80% of Dsg1 and Dsg3 were detected in the detergent-insoluble pool (Figure 6b), suggesting that this fraction is associated with desmosomes. Interestingly, Flot1 also exhibited an equally high degree of detergent insolubility, whereas Flot2 was equally distributed between detergent soluble and insoluble fractions. Please note that 7.5% of the soluble pools were loaded onto the gel, whereas the insoluble samples represent only 0.93% of the total insoluble fraction! The different percentages have been taken into account in the quantification shown in Figure 6b. 

**Figure 7:**
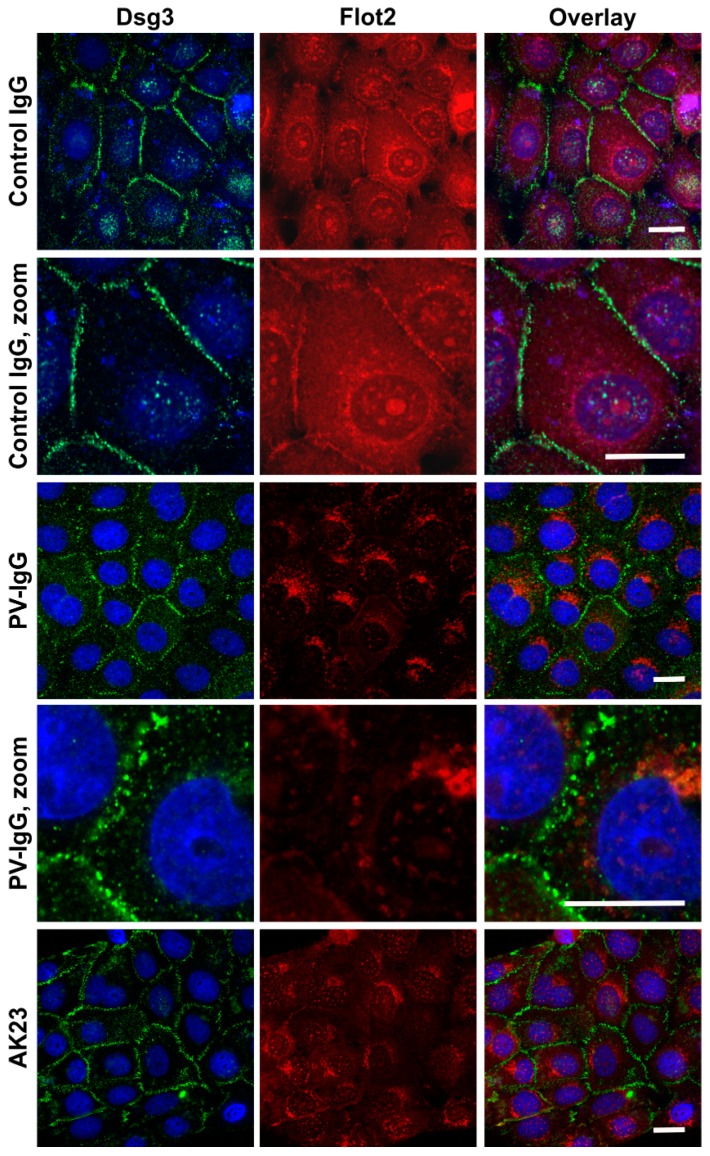
Effect of purified Pemphigus auto-antibodies (PV-IgG) and anti-Dsg3 antibodies on the localization of Dsg3 and Flot2 in hTert cells. The cells were grown on coverslips in KGM with 0.05 mM calcium for at least four days, and then shifted to 2 mM calcium for 24 h. Thereafter, the cells were treated with either purified control Immunoglobulin G (IgG), PV-IgG or pathogenic monoclonal anti-Dsg3 (AK23) antibody for further 24 h. After MeOH fixation, the cells were stained with anti-Dsg3 (green) and anti-Flot2 (red) antibodies and fluorochrome coupled secondary antibodies (anti-mouse Alexa488 and anti-rabbit Alexa546). The coverslips were mounted in a mounting medium with DAPI. *n* = 3 independent experiments, scale bar 20 µm.

Treatment of keratinocyte monolayers with purified antibodies from PV patients (PV-IgG) or with the highly pathogenic monoclonal anti-Dsg3 antibody AK23 results in acantholysis and disruption of desmosomal cell adhesion [10]. hTert monolayers were grown for 24 h under 2 mM calcium, after which they were treated for further 24 h under 2 mM calcium with 150 µg/mL purified Control IgG or PV-IgG from a PV patient or with 10 µg/mL purified AK23 antibody. Immunostaining for Dsg3 and Flot2 was performed after the treatment and fixation. Figure 7 shows that both PV-IgG and AK23 resulted in an altered staining pattern of Dsg3 as compared to control IgG treated cells. The continuous staining of Dsg3 at cell-cell borders was disrupted into a dot-like pattern (Figure 7), indicating that desmosomal organization was disturbed. In cells treated with control IgG, Flot2 was partially colocalized with Dsg3 at cell-cell borders. However, after PV-IgG or AK23 treatment, Flot2 was found almost exclusively in intracellular, perinuclear regions, and no colocalization with Dsg3 was detected, similarly to what we have previously shown in HaCaT keratinocytes [9]. 

**Figure 8:**
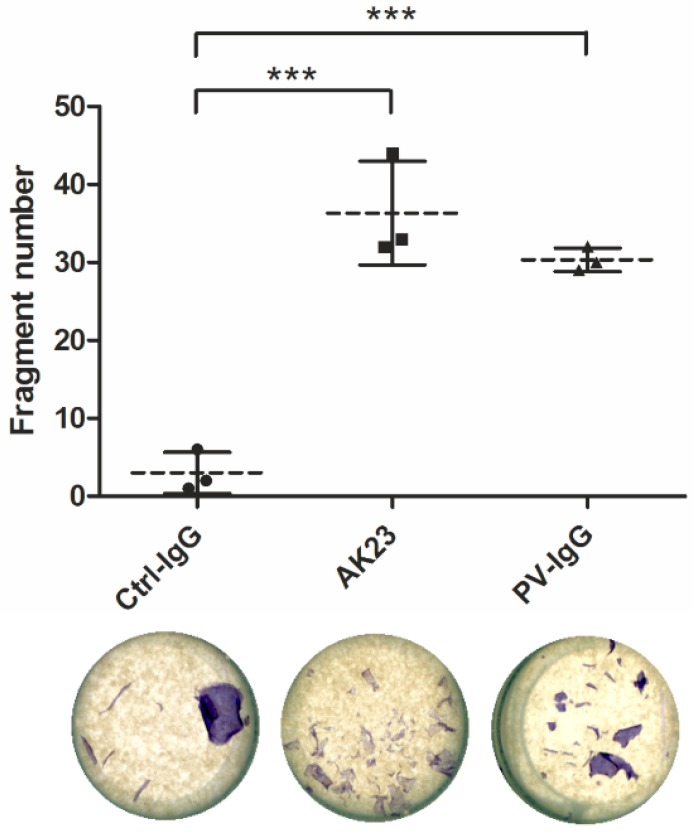
Effect of PV-IgG and anti-Dsg3 antibodies on the monolayer integrity in hTert cells. The cells were grown in KGM with 0.05 mM calcium until confluent and then shifted to 2 mM calcium for 24 h. Thereafter, the cells were treated with either purified control IgG, PV-IgG or AK23 antibody for further 24 h. Dispase was used to break the cell-matrix adhesion, and resulting monolayers were pipetted up and down to induce fragmentation. The resulting fragments were counted using ImageJ. Statistical analysis was done using ANOVA with Bonferroni’s multiple comparison test. Statistically significant differences are indicated by *** *p* < 0.001. *n* = 3 independent experiments. Dotted line: Mean of samples, solid lines: SD.

Cell-cell adhesion strength after PV-IgG treatment can be measured by dissociating the cell monolayer from the plate surface by treatment with Dispase, an enzyme that breaks the cell-matrix contacts but leaves cell-cell adhesion intact [16]. Monolayers of confluent hTert cells were treated with control IgG, AK23 or PV-IgG prior to treatment with Dispase. The monolayers were disrupted by pipetting, with all samples treated in an identical way, and the resulting fragments were stained and counted (Figure 8). Control IgG treated cells exhibited only very few fragments, whereas AK23 and PV-IgG treated monolayers were dissociated to 30–40 fragments (Figure 8), demonstrating that both AK23 and PV-IgG induced a profound weakening of the desmosomal adhesion in hTert cells. 

## 3. Discussion

We here have tested the suitability of hTert cells as a model system for the research on desmosomal adhesion and the molecular pathomechanisms of Pemphigus. The expression of desmogleins was found to be dependent on calcium concentration and thus on the maturation of desmosomes. This appears to be based on increased protein expression or reduced degradation, whereas transcriptional upregulation does not seem to play a major role. High level of Dsg expression was only observed upon high calcium, and the localization of desmogleins was altered upon low (intracellular) vs. high calcium (plasma membrane) conditions. This provides an additional advantage for studies on desmosomal adhesion, as the formation of desmosomes can be modulated or even induced by a calcium switch, similarly to primary keratinocytes (see e.g., [17,18]). Such dynamic studies are useful for studying factors such as flotillins that may play a role in regulating desmosomal dynamics [9,19]. 

In dense monolayer cultures of hTert cells grown with 2 mM calcium for 24 h, more than 80% of Dsg1 and Dsg3 are found in the detergent insoluble fraction, which is considered to represent the pool of these proteins that is associated with mature desmosomes. This is well comparable to HaCaT cells grown as dense monolayers under high calcium conditions [9]. Here, we did not perform a direct comparison of detergent-insolubility of Dsgs in low vs. high calcium, since the expression levels are 3–5-fold different, which would complicate the comparison due to unequal protein amounts. However, since Dsgs are intracellularly localized upon low calcium, as we have shown here, it is highly likely that they mainly reside in the soluble, non-desmosomal pool. 

In Pemphigus, autoantibodies against desmosomal proteins, mainly Dsg3 and Dsg1, result in weakening of the adhesion between keratinocytes. Experimentally, this can be tested by a cell monolayer fractionation assay after treatment of the cells with antibodies against Dsg3 and enzymatic disruption of cell-matrix adhesion by Dispase [16]. We here show that treatment of hTert cell monolayers with either a monoclonal anti-Dsg3 antibody AK23 or purified IgG fractions from PV patients results in a much larger number of fragments than treatment with control IgGs from healthy persons. These data show that hTert cells are well suitable for functional studies of PV-IgG or anti-Dsg mediated effects on keratinocyte adhesion. 

We have earlier shown that fotillins interact with desmogleins, and that Dsg3 localization is altered upon flotillin depletion [9]. Interestingly, in hTert cells that were cultured under high calcium, especially Flot2 underwent a change in its localization that is similar to the translocation of Dsg3 to the plasma membrane, but the expression level of Flot2 was not increased in 2 mM calcium. On the contrary, both the mRNA and protein levels of flotillins were either significantly reduced or showed a tendency to be reduced (Flot2 protein level). Upon calcium switch, a fraction of especially Flot2 was translocated from intracellular vesicles towards the plasma membrane, where a colocalization with Dsg3 was observed. These findings strongly point to a role for Flot2 in the formation of cell-cell contacts. Previous findings from us and others have shown that flotillins are also associated with cell adhesion mediated by further members of the cadherin protein family, such as E-cadherin [9,19,20,21]. Therefore, flotillins may be more general “cadherin partners” than previously presumed. 

We have postulated that flotillins may play a role in the regulation of desmosomal assembly and/or disassembly [9], which is supported by the data of the present study. However, it is interesting that although Flot1 shows a similar degree of detergent-insolubility as the desmogleins, Flot2 is about equally distributed between detergent-soluble and insoluble pools upon high calcium. This may point to individual functions of flotillins in the regulation of desmosomal adhesion. Although a large fraction of flotillins is observed as hetero-oligomers with each other, also homo-oligomers are detected [22]. We have shown that hetero-oligomerization of flotillins is required for the endocytosis of Flot2 from the plasma membrane upon EGF stimulation [22,23]. Therefore, dynamic changes in the degree of hetero-oligomerization of flotillins during desmosomal maturation or disassembly may be a mechanism to regulate Dsg localization. Our earlier data have shown that in flotillin knockdown HaCaT cells, Dsg3 expression is reduced due to its increased endocytosis and lysosomal degradation [9]. Thus, it will be interesting to study how various flotillin mutants that exhibit an altered oligomerization and cellular localization, such as the Tyr163Phe-Flot2 [22,23] affect desmosomal adhesion and desmoglein localization. 

In summary, we have here shown that the hTert/KER-CT cells exhibit a calcium-dependent expression and localization of desmogleins. Furthermore, treatment with PV-IgG or anti-Dsg3 antibody AK23 results in disruption of cell-cell adhesion and changes in Dsg3 and Flot2 localization. These findings well recapitulate those that we and others have previously presented for HaCaT cells that are the most popular cells used for studies of epidermal cell adhesion and Pemphigus mechanisms. Due to the clear advantages of hTert cells over HaCaT cells and primary keratinocytes, hTert cells can provide a valid and more versatile cell model for studies on desmosomal adhesion, Pemphigus pathogenesis and even for other studies in the field of dermatology in general. 

## 4. Materials and Methods 

### 4.1. Cell Culture

hTert/KER-CT cells (ATCC^®^, CRL4048™, Wesel, Germany) were cultured in Keratinocyte Growth Medium 2 (KGM2, PromoCell, Heidelberg, Germany) supplemented with 4 µL/mL Bovine Pituitary Extract, 0.125 ng/mL Epidermal Growth Factor (recombinant human), 5 µg/mL Insulin (recombinant human), 0.33 µg/mL Hydrocortisone, 0.39 µg/mL Epinephrine, 10 µg/mL Transferrin (recombinant human), 0.05 mM CaCl_2_ (PromoCell, Heidelberg, Germany) and 30 µg/mL Gentamycin sulphate (Serva, Heidelberg, Germany) at 37 °C and 5% CO_2_. For analysis under high calcium conditions (2 mM), the cells were grown in KGM2 with 2 mM CaCl_2_ as indicated prior to harvest. 

### 4.2. Antibodies

Mouse monoclonal antibodies (sc-137164, sc-23911, sc-23912, Santa Cruz, Heidelberg, Germany) were used for detection of desmoglein-1, desmoglein-2 and desmoglein-3 in Western blots and for immunofluorescence. Mouse monoclonal antibodies against flotillin-1 and flotillin-2 (BD Biosciences Heidelberg, Germany) were used for Western blots and immunofluorescence. A rabbit polyclonal antibody directed against flotillin-2 (F1680, Sigma Aldrich, Taufkirchen, Germany) was utilized for immunofluorescence double stainings with Dsgs. A mouse monoclonal GAPDH antibody was obtained from Abcam (ab8245, Cambridge, UK). The primary antibodies used for immunofluorescence were detected with an Alexa Fluor 488-conjugated donkey anti-mouse antibody (A10040, Invitrogen, Karlsruhe, Germany) and an Alexa Fluor 546 donkey anti-rabbit antibody (A21202, Invitrogen, Karlsruhe, germany). The primary antibodies used for Western blotting were detected with an HRP-conjugated goat anti-mouse antibody (Dako, Glostrup, Denmark). 

### 4.3. Cell Lysis, Gel Electrophoresis and Western Blot Analysis

Cells were scraped into lysis buffer (50 mM Tris pH 7.4; 150 mM NaCl; 2 mM EDTA; 1% NP-40) supplemented with protease inhibitor cocktail (Sigma Aldrich, Taufkirchen, Germany) and incubated on ice for 30 min. The cell lysates were cleared by centrifugation (15,000 rpm, 4 °C, 10 min) and the protein amount was measured using the Bradford assay (Bio-Rad, Munich, Germany). Equal protein amounts were analyzed by SDS-PAGE and Western blot. 

### 4.4. Immunofluorescence

3 × 10^4^ hTert cells were seeded on glass coverslips and cultured for at least four days in KGM2 with 0.05 mM CaCl_2_. For high calcium conditions, the medium was exchanged to KGM2 with 2 mM CaCl_2_ 24 h prior to fixation. All cells were fixed with methanol for 8 min at –20 °C. Subsequently, the cells were blocked with 1% BSA for 30 min and staining with primary and secondary antibodies was carried out for 1 h at room temperature in 1% BSA/PBS. Samples were analyzed using a Zeiss LSM710 Confocal Laser Scanning Microscope (Carl Zeiss, Oberkochen, Germany). 

### 4.5. PV-IgG and AK23 Purification and Treatment of hTert Keratinocytes

Serum samples were obtained from patients with verified diagnosis of Pemphigus vulgaris, and the IgG fraction was purified as described previously [24]. The AK23 hybridoma cell line [10] was cultured as a suspension in RPMI, 10% FSC, non-essential amino acids, pyruvate and Penicillin/Streptomycin. In addition, 55 µM β-mercapto-ethanol was added to the medium. For antibody production, the cells were seeded into a medium containing 40% of the culture medium and 60% of ISF-I hybridoma medium (Biochrom, Berlin, Germany) and grown for six days. The antibody fraction was purified using Protein G Sepharose 4 Fast Flow (Sigma Aldrich, Taufkirchen, Germany) and concentrated in PBS. 

Keratinocytes were grown to 90% confluence in KGM2 with 0.05 mM CaCl_2_, prior to an incubation in KGM2 with 2 mM CaCl_2_ for 24 h. The cells were treated with either 150 µg/mL purified PV-IgG, 150 µg/mL control-IgG from a healthy person or 1:100 AK23 purified hybridoma supernatant (1 mg/mL) for 24 h in 2 mM CaCl_2_ medium at 37 °C and 5% CO_2_. 

### 4.6. RNA Isolation and cDNA Synthesis

For RNA isolation, cells were solubilized in 1.6 mL Trizol (Invitrogen, Karlsruhe, Germany) for 5 min at room temperature before partitioning the samples equally. After addition of 200 µL chloroform, samples were vortexed for 10 s and allowed to rest for 5 min at room temperature, followed by a centrifugation step at 12,000 g for 15 min at 4 °C. The supernatant (upper clear phase) was transferred to a new tube. 400 µL isopropanol was added, and the tubes were inverted several times before another rest at room temperature for 10 min. Subsequently, the samples were centrifuged again at 12,000 g for 10 min at 4 °C. The supernatant was discarded, the pellet resolved in 75% ethanol and mixed by vortexing, followed by another centrifugation step at 7500 g for 5 min at 4 °C. The supernatant was again discarded and the ethanol wash was repeated prior to centrifugation at 7500 g for 5 min at 4 °C. The pellet was air-dried for 30 min before dissolving in 25 µL DEPC. Subsequently RNA concentrations were measured using the Tecan infinite M200 reader (Thermo Fisher, Karlsruhe, Germany). 

### 4.7. Quantitative Real-Time PCR

For qPCR, 3 µg of total RNA was reverse-transcribed using the M-MuLV reverse transcriptase (NEB, Frankfurt, Germany), the M-MuLV buffer and 150 fmol oligo(dT) primers in a total volume of 45 µL. qRT-PCR was performed using the CFX Connect Real-Time OCR Detection System (Bio-Rad, Munich, Germany). Annealing temperature was 60 °C for all primers. Samples were analyzed as duplicates using 0.2 µL of 10-fold diluted cDNA in a final volume of 10 µL with iTaqTMUniversal SYBR Green Supermix (Bio-Rad, Munich, Germany). The ∆Ct-method was used to quantify the PCR products. The geometric mean of the reference genes *RPL13a*, *GAPDH*, *Ywhaz* and *B2M* was used for normalization. The primer sequences are listed in Table 2.

### 4.8. Sequential Detergent Extraction 

Sequential detergent extraction was performed according to Stahley et al. [15]. hTert keratinocytes were grown until confluent, cultivated in 2 mM CaCl_2_ for 24 h, washed with cold PBS and incubated with 200 μL Triton buffer (1% Triton X-100; 10 mM Tris-HCl pH 7.5; 140 mM NaCl; 5 mM EDTA; 1 mM PMSF; 1 µg/mL Leupeptin; 1 µg/mL Pepstatin A) for 10 min on ice. The cells were scraped, vortexed for 30 s and centrifuged at 14,000 × g for 30 min. The supernatant containing the Triton soluble proteins was kept. The pellets (Triton insoluble proteins) were subsequently extracted with 400 μL of SDS-Urea buffer (1% SDS; 8 M urea; 10 mM Tris-HCl pH 7.5; 140 mM NaCl; 5 mM EDTA; 2 mM EGTA). 15 μL of the Triton soluble samples and 3.75 µL of the Triton insoluble samples were analyzed by SDS-PAGE and Western blot. The Triton X-100 insoluble pool has double the final volume compared to the Triton X-100 soluble pool. This was taken into account in the quantitative analysis by multiplying the signals of the insoluble pool by eight.

### 4.9. Dispase Based Dissociation Assay

Cells were seeded in a 24 well plate and grown until confluent. Upon confluence, KGM2 (0.05 mM CaCl_2_) was exchanged to KGM2 with 2 mM CaCl_2_ for 24 h. Cells were treated with either AK23 (75 µg/mL), PV-IgG (0.5 mg/mL) or control-IgG (0.3 mg/mL) for further 24 h at 37 °C and 5% CO_2_. The cells were washed with HBSS (Cat. 14025-050, Gibco, Karlsruhe, Germany) and incubated with 2.5 U/mL Dispase II (Cat. 04942078001, Roche, Mannheim, Germany) solution until the monolayers were completely detached. Subsequently, the cells were incubated with MTT (5 mg/mL) for 15 min. For fragmentation, mechanical stress was applied to the monolayer by pipetting up and down. The same conditions were used for all samples. The fragments were fixed using 4% paraformaldehyde and counted automatically using the imageJ software.

### 4.10. Data Quantification and Statistical Analysis

All experiments were performed at least three times, as indicated in the figure legends. For quantification and statistical analysis, Western blot bands of proteins were quantified by scanning densitometry using the Quantity-One software (BioRad, Munich, Germany), and the signals were normalized to GAPDH. The data are expressed as mean ± SD. For comparison of two independent groups, unpaired Student’s *t*-test was used. For comparison of three independent groups, one-way analysis of variance (ANOVA) with Bonferroni’s multiple comparison test was used. Statistically significant differences are indicated in the figures by * *p* < 0.05, ** *p* < 0.01 and *** *p* < 0.001. 

### 4.11. Electronic Manipulation of Images

The fluorescence images have in some cases as a whole been subjected to brightness or contrast adjustments. No other manipulations of images have been performed, unless otherwise stated.

### 4.12. Ethical Statement

This study was approved by the Ethics Committee of the Medical Faculty of the Philipps-Universität, Marburg (Registry number 20/14).

## Figures and Tables

**Table 1:** Comparison of the three most common cell models used for studies in Pemphigus.

Features	HaCaT	Primary Keratinocytes	hTert Cells
Origin	Pre-lesional skin	Mainly Foreskin	Foreskin
Immortal growth	Yes	No	Yes
Chromosomal aberrations	Numerous	?	Trisomy 5 and 20
Ca-dependent differentiation	Partly	Yes	Yes
3D cultures	Partly	Yes	Yes
Specific features	Inactive p53 protein	Varying genotype (several donors)	Express telomerase and Cdk4
Susceptible to genetic modifications (CRISPR/Cas)	Yes	No	Yes

**Table 2:** The qPCR primers used in this study with their sequences.

Target Gene	Primer Name	Sequence 5′-3′
*Dsg1*	Dsg1-fwd	TGAACCCGGAAACGGAGCCA
	Dsg1-rew	AAGCCAGCTGCACTACGAGGA
*Dsg3*	Dsg3-fwd	CAACCCTCAATGCTACCTCGGC
	Dsg3-rev	AGACTTCCAGTGTCAAGCTGCG
*Dsc1a*	Dsc1a-fwd	GCTTGGCGAAAAGGTGTATTTG
	Dsc1a-rev	AACTCCAGTCCCTCTTCTTCC
*Dsc1b*	Dsc1b-fwd	CTTGGAGTCCGTCAAGGGAGTG
	Dsc1b-rev	TAATGGATTCTTCGCCAAGCCGAG
*Flot1*	Flot-1-fwd	TATGCAGGCGGAGGCAGAAG
	Flot-1-rev	CAGTGTGATCTTATTGGCTGAA
*Flot2*	Flot-2-fwd	GAGATTGAGATTGAGGTTGTG
	Flot-2-rev	ATCCCCGTATTTCTGGTAGG
*E-cadherin*	E-cad-fwd	ACCCTGGTGGTTCAAGCTGCTG
	E-cad-rev	CTGACCCTTGTACGTGGTGGGA
*γ-catenin*	γ-cat-fwd	GATGGCCCAGAACTCTGTGCGT
	γ-cat-rev	AGCCGATGGTTGCCTTGACCAG
*GAPDH*	GAPDH-fwd	CATCTTCCAGGAGCGAGATCCC
	GAPDH-rev	CCAGCCTTCTCCATGGTGGT
*RPL13a*	RPL13a-fwd	CCTGGAGGAGAAGAGGAAAGAGA
	RPL13a-rev	TTGAGGACCTCTGTGTATTTGTCAA
*B2M*	B2M-fwd	AGATGAGTATGCCTGCCGTGTG
	B2M-rev	TGCGGCATCTTCAAACCTCCA
*Ywhaz*	Ywhaz-fwd	AGGTTGCCGCTGGTGATGAC
	Ywhaz-rev	GGCCAGACCCAGTCTGATAGGA

## Supplementary Materials

Supplementary materials can be found at https://www.mdpi.com/1422-0067/20/13/3113/s1.

## Abbreviations

AK23	Pathogenic monoclonal anti-Dsg3 antibody
ANOVA	Analysis of variance
B2M	Beta-2-microglobulin
BSA	Bovine serum albumin
CaCl_2_	Calcium chloride
Cdk4	Cyclin-dependent kinase 4
CRISPR	Clustered Regularly Interspaced Short Palindromic Repeats
DAPI	4′,6-diamidino-2-phenylindole
DEPC	Diethyl pyrocarbonate
DOAJ	Directory of open access journals
Dsc	Desmocollin
Dsg	Desmoglein
EDTA	Ethylenediaminetetraacetic acid
EGF	Epidermal growth factor
EGTA	Ethylene glycol-bis(β-aminoethyl ether)-*N*,*N*,*N*′,*N*′-tetraacetic acid
FCS	Fetal calf serum
Flot1	Flotillin-1
Flot2	Flotillin-2
GAPDH	Glyceraldehyde 3-phosphate dehydrogenase
HaCaT	Human adult high Calcium low Temperature
HBSS	Hank’s Balanced Salt Solution
HRP	Horseradish peroxidase
hTert	Human Telomerase reverse transcriptase
IgG	Immunoglobulin G
KGM	Keratinocyte Growth Medium
MDPI	Multidisciplinary Digital Publishing Institute
MeOH	Methanol
MTT	3-(4,5-dimethylthiazol-2-yl)-2,5-diphenyltetrazolium bromide
NaCl	Sodium chloride
NP-40	Nonyl phenoxypolyethoxylethanol
PAGE	Polyacrylamide gel electrophoresis
PBS	Phosphate buffered saline
PF	Pemphigus foliaceus
PMSF	Phenylmethylsulfonyl fluoride
PV	Pemphigus vulgaris
qPCR	Quantitative polymerase chain reaction
RPL13a	60S ribosomal protein L13a
SD	Standard deviation
SDS	Sodium dodecyl sulfate
TX	Triton X-100
Ywhaz	14-3-3 protein zeta/delta
