# Supplementary material for: Immortalized Human hTert/KER-CT Keratinocytes a Model System for Research on Desmosomal Adhesion and Pathogenesis of Pemphigus Vulgaris
Source: Int J Mol Sci. 2019 Jun 26;20(13):3113. doi: 10.3390/ijms20133113 (PMC6651391; doi:10.3390/ijms20133113)

Supplementary Figures ijms-532768

Full Blots for Figure 4. For Dsg blots, the upper part of the gel (MW range above 60 kDa) was used, whereas the lower part was used for Flots. GAPDH was incubated on the same blot slice as Flot2.

Dsg1 **Dsg2** **Dsg3** **Flot1**

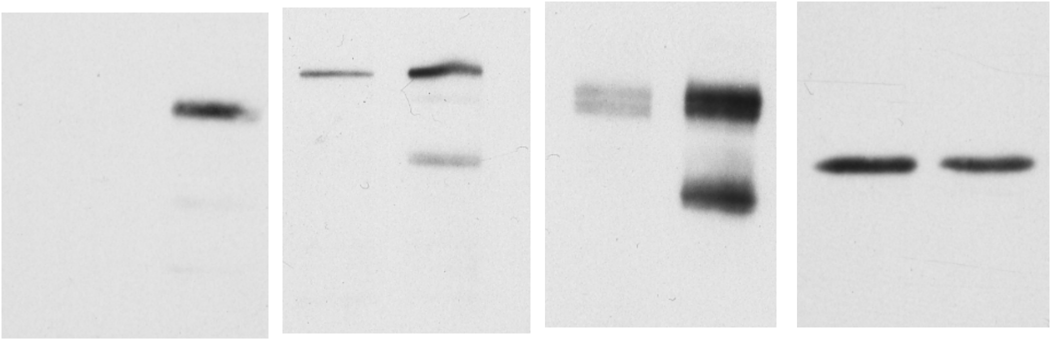

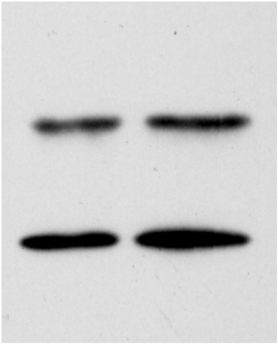

Flot2

GAPDH

Full Blots for Figure 6. For Dsg blots, the upper part of the gel (MW range above 60 kDa) was used, whereas the lower part was used for Flots.

Dsg1 **Dsg3** **Flot1** **Flot2**

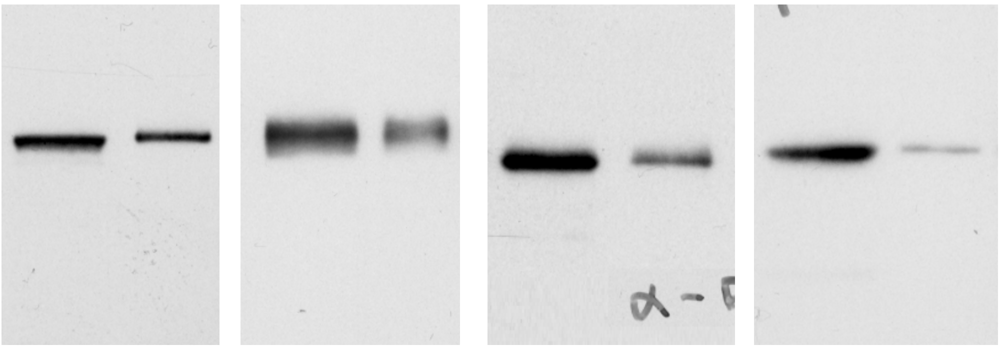

Supplement: Supplementary file 1 [file ijms-20-03113-s001.pdf]
